# Supplementary material for: Gut-Testis Axis: Microbiota Prime Metabolome To Increase Sperm Quality in Young Type 2 Diabetes
Source: Microbiol Spectr. 2022 Oct 10;10(5):e01423-22. doi: 10.1128/spectrum.01423-22 (PMC9603910; doi:10.1128/spectrum.01423-22)
Supplement: Supplemental file 1 — Supplemental material. Download spectrum.01423-22-s0001.pdf, PDF file, 2.8 MB [file spectrum.01423-22-s0001.pdf]

Supplementary information

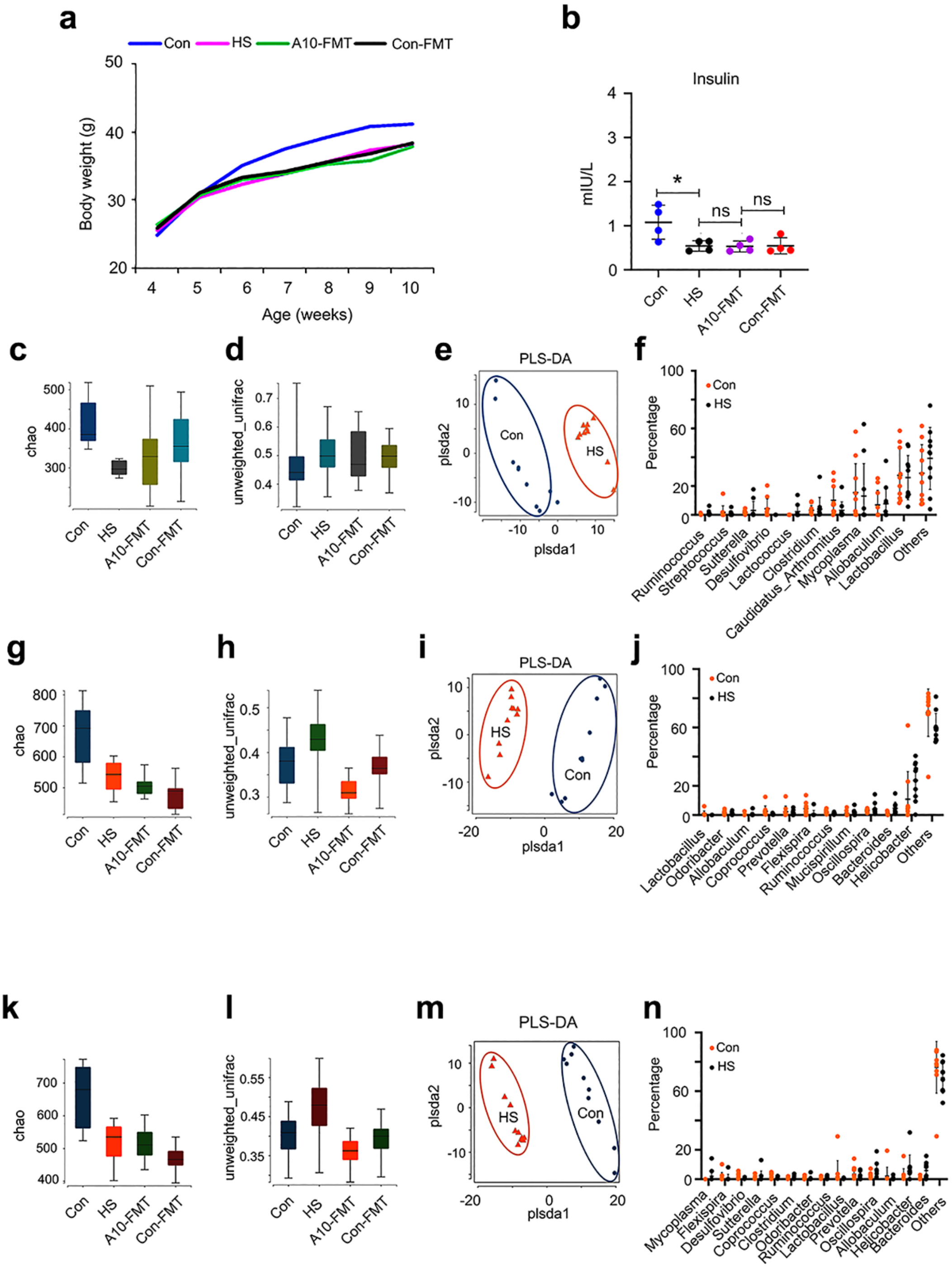

Fig. S1. Body weight and gut microbiota changes (HS vs. Con). **a** Animal bodyweight. The y-axis represents the body weight (g). The x-axis represents the age (weeks). **b** Blood insulin levels. The y-axis represents the concentration (mIU/L). The x-axis represents the treatment. **c** The alpha index of the small intestine microbiota (Chao index). The y-axis represents the relative amount. The x-axis represents the treatment. **d** The beta index of small intestinal microbiota. The y-axis represents the relative amount. The x-axis represents the treatment. **e** PLS-DA (OTU) of small intestine microbiota in HS and Con groups. **f** Small intestine microbiota levels at the genus level in HS and Con groups. The y-axis represents the relative amount (%). The x-axis represents the individual microbiota. **g** The alpha index of the cecum

microbiota (Chao index). The y-axis represents the relative amount. The x-axis represents the treatment. **h** The beta index of cecum microbiota. The y-axis represents the relative amount. The x-axis represents the treatment. **i** PLS-DA (OTU) of cecum microbiota in HS and Con groups. **j** Cecum microbiota levels at the genus level in HS and Con groups. The y-axis represents the relative amount (%). The x-axis represents the individual microbiota. **k** The alpha index of the colon microbiota (Chao index). The y-axis represents the relative amount. The x-axis represents the treatment. **l** The beta index of colon microbiota. The y-axis represents the relative amount. The x-axis represents the treatment. **m** PLS-DA (OTU) of colon microbiota in HS and Con groups. **n** Colon microbiota levels at the genus level in HS and Con groups. The y-axis represents the relative amount (%). The x-axis represents the individual microbiota.

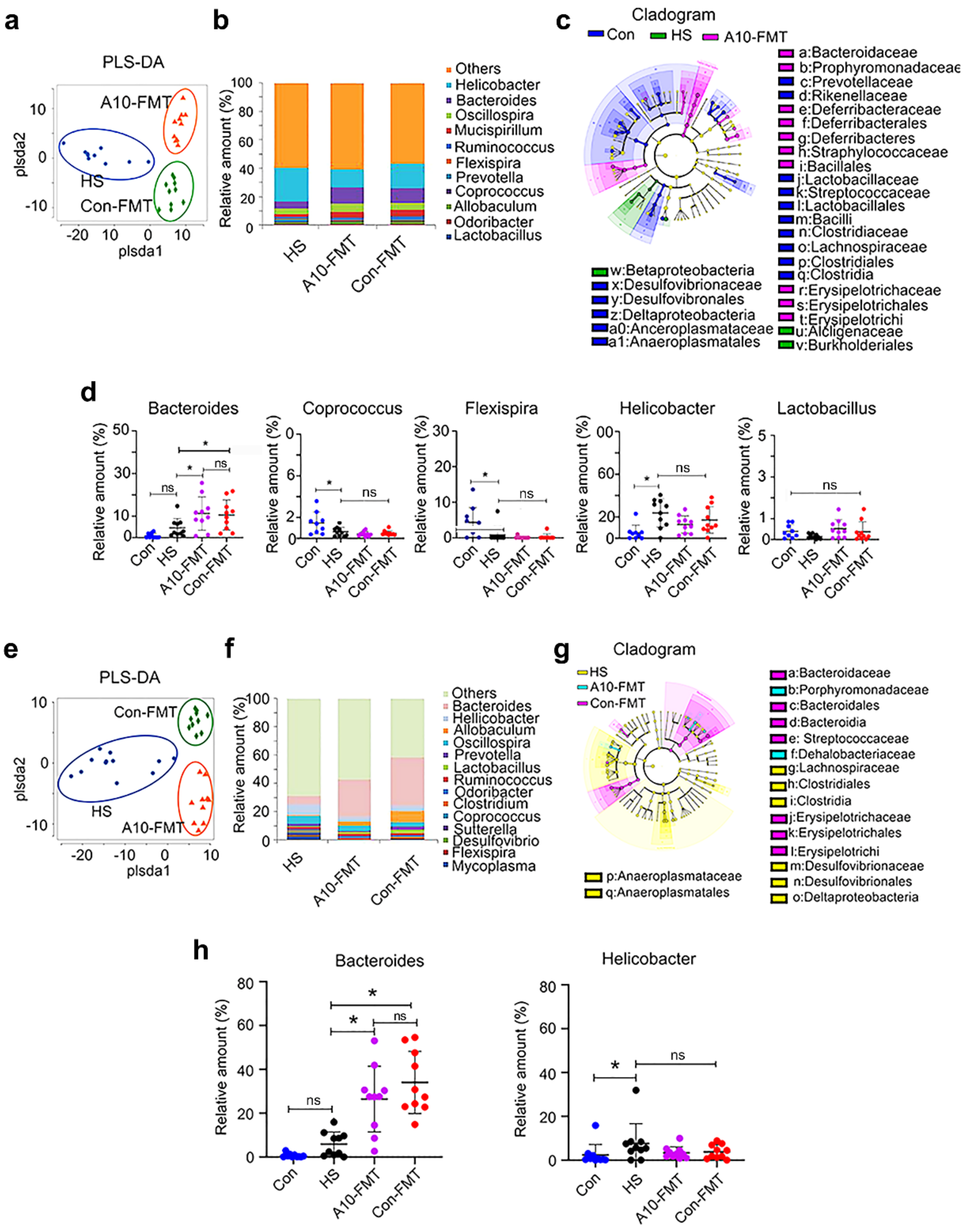

Fig. S2. A10-FMT improved cecal and colon microbiota in type 2 diabetes. a PLS-DA (OTU) of cecum microbiota in HS, A10-FMT, and Con-FMT groups. **b** Cecum microbiota levels at the genus level in HS, A10-FMT, and Con-FMT groups. The y-axis represents the relative amount (%). The x-axis represents the treatments. Different colors represent different microbiota. **c** Cladogram of the LEfSe determining the cecum microbiota difference in abundance. **d** *Bacteroides, Mucispirillum, Coprococcus, Flexispira, Helicobacter, Lactobacillus* in the cecum. The y-axis represents the relative amount at the genus level. The x-axis represents the treatment. *$p < 0.05$. **e** PLS-DA (OTU) of colon microbiota in HS, A10-FMT, and Con-FMT groups. **f** Colon microbiota levels at the genus level in HS, A10-FMT, and Con-FMT groups. The y-axis represents the relative amount (%). The x-axis represents the treatments. Different colors represent different microbiota. **g** Cladogram of the linear discriminate analysis effect size (LEfSe) determining the difference in abundance of small intestine microbiota. **h** *Bacteroides, Helicobacter* in the colon. The y-axis represents the

relative amount at the genus level. The x-axis represents the treatment. *$p < 0.05$.

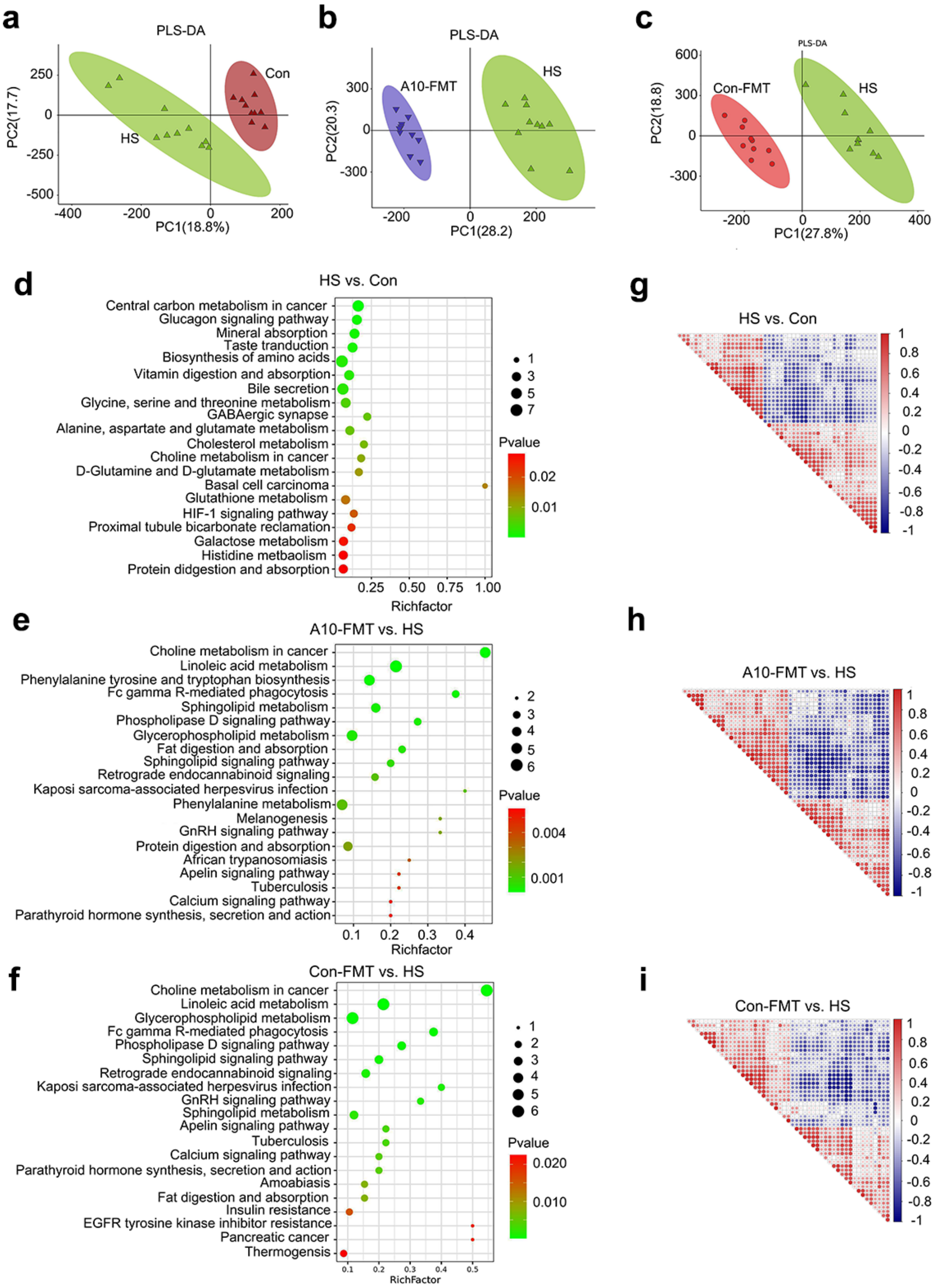

Fig. S3. Blood metabolic data by LC/MS. **a** PLS-DA of blood metabolites in the HS and Con groups. **b** PLS-DA of blood metabolites in the HS and A10-FMT groups. **c** PLS-DA of blood metabolites in the HS and Con-FMT groups. **d** Enriched pathways of changed blood metabolites in HS vs. Con. **e** Enriched pathways of changed blood metabolites in A10-FMT vs. HS. **f** Enriched pathways of changed blood metabolites in Con-FMT vs. HS. **g** Correlation of changed metabolites themselves in HS vs. Con. **h** Correlation of changed metabolites themselves in A10-FMT vs. HS. **i** Correlation of changed metabolites themselves in Con-FMT vs. HS.

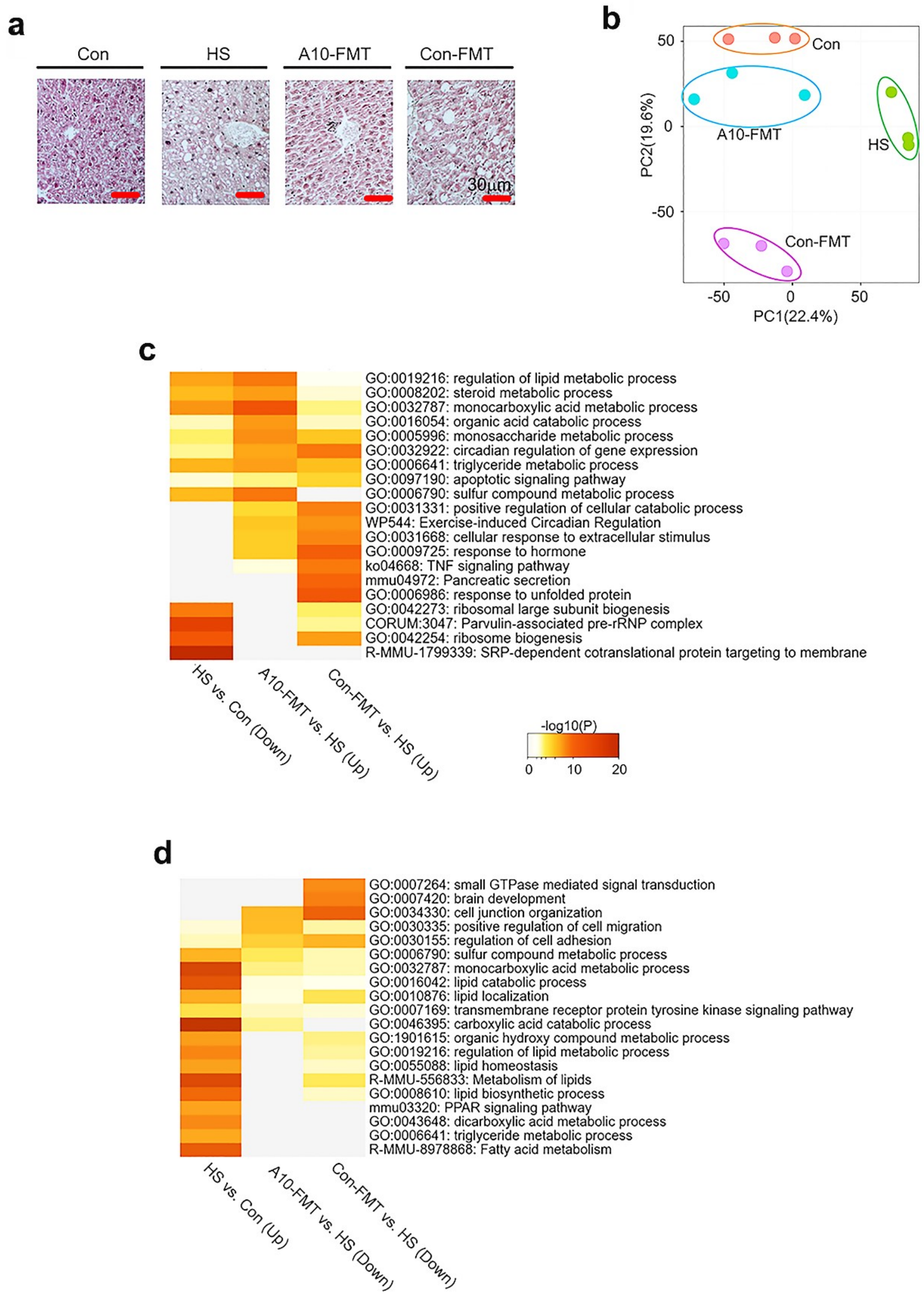

Fig. S4. A10-FMT improved liver function. **a** Histopathology analysis (HE) of liver. **b** The PCA of liver gene expression data. **c** The functional enrichment analysis of HS decreased genes while these were increased by A10-FMT or Con-FMT in the liver. **d** The functional enrichment analysis of HS increased genes while these were decreased by A10-FMT or Con-FMT in the liver.

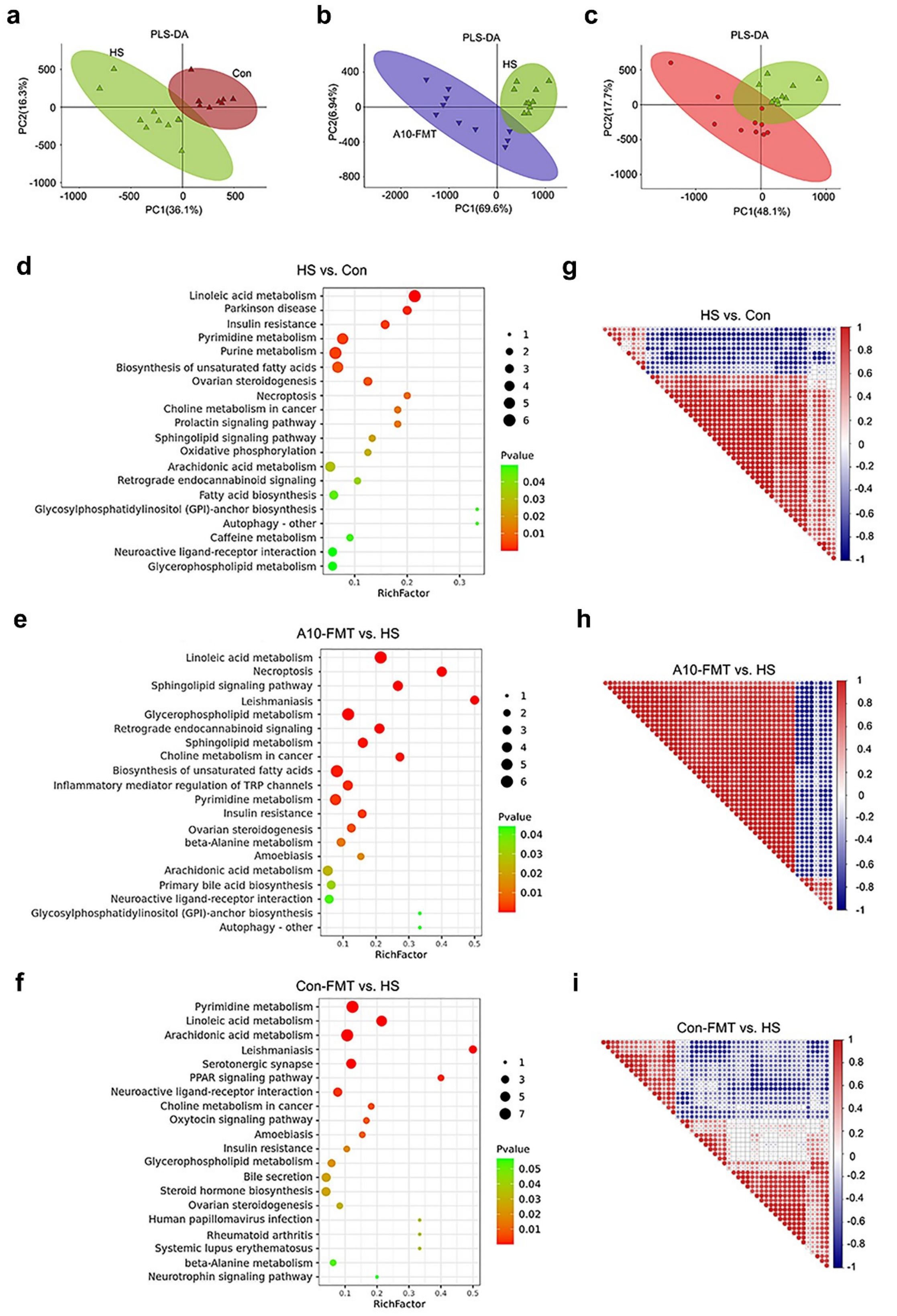

Fig. S5. Testicular metabolic data by LC/MS. **a** PLS-DA of testicular metabolites in the HS and Con groups. **b** PLS-DA of testicular metabolites in the HS and A10-FMT groups. **c** PLS-DA of testicular metabolites in the HS and Con-FMT groups. **d** Enriched pathways of changed testicular metabolites in HS vs. Con. **e** Enriched pathways of changed testicular metabolites in

A10-FMT vs. HS. **f** Enriched pathways of changed testicular metabolites in Con-FMT vs. HS. **g** Correlation of testicular changed metabolites themselves in HS vs. Con. **h** Correlation of testicular changed metabolites themselves in A10-FMT vs. HS. **i** Correlation of testicular changed metabolites themselves in Con-FMT vs. HS.

Table S1. Primary antibody information.

Gene symbol	Name	Cat. #	Predicted size	Source (Animal)	Company
DDX4 (VASA)	DEAD (Asp Glu Ala Asp) box polypeptide	ab13840	76kDa	Rabbit (polyclonal)	Abcam
Bax	BCL2-Associated X	bs-4564R	21kd	Rabbit (polyclonal)	Beijing Biosynthesis Biotechnology CO.
SCP3/SYCP3	Synaptonemal complex protein 3	NB300-232	28kDa	Rabbit (polyclonal)	Novus Biologicals
SOX9	SRY (sex-determining region Y)-box 9 protein	AB5535	65kDa	Rabbit (polyclonal)	Merck Millipore
TNP1(TP1)	Transition protein-1	ab73135	6.4kDa	Rabbit (polyclonal)	Abcam
Bcl-2	Bcl-2	bs-4563R	26kd	Rabbit (polyclonal)	Beijing Biosynthesis Biotechnology CO.
CYP11A1	Cholesterol side chain cleavage enzyme	bs-10099R	53/57kDa	Rabbit (polyclonal)	Beijing Biosynthesis Biotechnology CO.
PIWIL1	Piwi like protein 1	ab94917	99kDa	Rabbit (polyclonal)	Abcam
Caspase 3	Caspas 3	bs-0081R	28kd	Rabbit (polyclonal)	Beijing Biosynthesis Biotechnology CO.
PGK2	Phosphoglycerate kinase 2	D121803	45kDa	Rabbit (polyclonal)	Sangon Biotech (Shanghai) Co., Ltd.
HSD17b1	Hydroxysteroid (17-beta) dehydrogenase 1	bs-3855R	35kd	Rabbit (polyclonal)	Beijing Biosynthesis Biotechnology CO.
ODF1	Outer defense fiber 1	sc-390152	27kd	Mouse (monoclonal)	Santa Cruz Biotechnology, Inc.
CYP7A1	Cholesterol 7 alpha hydroxylase	bs-21429R	55kd	Rabbit (polyclonal)	Beijing Biosynthesis Biotechnology CO.
DHRS9	Dehydrogenase/reductase SDR family member 9	bs-7859R	35kDa	Rabbit (polyclonal)	Beijing Biosynthesis Biotechnology CO.
RBP4	Retinol binding protein 4	D122994-0025	23kDa	Rabbit (polyclonal)	Sangon Biotech (Shanghai) Co., Ltd.
NR1H4	Bile Acid Receptor (NR1H4)	Bs-12867R	56kDa	Rabbit (polyclonal)	Beijing Biosynthesis Biotechnology CO.
NR1H3	LXR alpha + LXR beta	bs-18451R	51kDa	Rabbit	Beijing Biosynthesis Biotechnology CO.
GPX1	Glutathione peroxidase 1	bs-3882R	22kDa	Rabbit (polyclonal)	Beijing Biosynthesis Biotechnology CO.
actin	actin	Ab3280	42kDa	Rabbit (polyclonal)	Abcam

Data Set 1. Blood metabolites raw data.

Data Set 2. Testicular metabolites raw data.
